# The Cost of Provisions in Institutions.—VIII

**Published:** 1904-06-11

**Authors:** 


### THE COST OF PROVISIONS IN INSTITUTIONS.—VIII.

By the Author of "Hospital Expenditure—The Commissariat."

(*Continued from page* 177.)

### UNIVERSITY COLLEGE HOSPITAL (Available Beds, 188).

The cost per bed in this hospital for provisions during the last three years is as follows:—

| Year | | Average of occupied beds |
|---|---|---|
| | £ | |
| 1902 ... ... ... ... | 30 | 168 |
| 1901 ... ... ... ... | 34½ | 164 |
| 1900 ... ... ... ... | 31½ | 162 |

Prior to 1900 the board of the nursing staff was not specified, being undertaken by the sisterhood and included in the charge for nursing. The cost of provisioning the hospital was thus apparently reduced to very small proportions—£18 per bed, in comparison with £30 at the present time. For the cost of boarding the nursing staff is at least one-third of the total cost of provisions for the institution.

In the published statement of accounts for 1902 the cost of provisions is given under two headings:—

(*a*) Provisions for the hospital, £3,435.
(*b*) Provisions for the nursing home, £1,595.

Under "Provisions for the hospital" is included the board of a daily average of 168 patients, 12 medical officers, and 23 female servants. In addition 6 lay officers receive luncheon, and 7 male servants receive dinner.

Under "Provisions for the nursing home," in 1902, is included the board of the nursing staff exclusively, numbering 85.

The accounts do not permit of ascertaining with rigid accuracy the cost of the patients' board. It is possible, however, to get an approximate estimate by eliminating the other items included with it. Analysis of the accounts has shown that the weekly cost of provisions for the medical officers' table is £1 1s. a head. The cost of the officers' board at this rate is £648 a year. Reckoning the additional lunches and dinners together at 9d. a head a day, the yearly cost is £177. Reckoning the board of the female servants at 6s. a week each, the yearly cost is £358. The total cost of those other than patients is then approximately £1,183, leaving £2,252 to be divided among a daily average of 168 patients. This works out at just over £13, or almost exactly 5s. a week.

The cost of provisions for the nursing staff averages £18 5s. a year, or exactly 7s. a week each.

It is interesting for purposes of comparison with other hospitals to work out the cost per bed of "persons other than patients," but the figures being based on an *approximate* estimate of the patients' cost have less value than would be the case if the accounts showed the actual expenditure on this item.

| Year | Average No. of occupied beds out of 188 available | Total cost of provisions | Cost of patients at £13 a year | Average cost of provisions, exclusive of patients | |
|---|---|---|---|---|---|
| | | | | Per available bed | Per occupied bed |
| | | £ | £ | | |
| 1902 | 168 | 5,030 | 2,184 | 15 | 17 |
| 1901 | 164 | 5,657 | 2,132 | 18½ | 20½ |
| 1900 | 162 | 5,086 | 2,106 | 15¾ | 18 |

The proportion of those boarded other than patients is 43 per cent. of the total inmates, the numbers being as follows:—

| — | Number | Average No. of available beds to each | Average of occupied beds to each |
|---|---|---|---|
| Medical officers... | 12 | 15·6 | 14 |
| Nursing staff ... | 85 | 2·2 | 2 |
| Female servants | 23 | 8 | 7 |
| Lunches and dinners equivalent to board of 6 extra persons... | 6 | — | — |
| Total ... | 126 | 1·5 | 1·3 |

The average cost per head of each item of provisions during the year can in the case of the nursing staff be exactly estimated, and it is as follows:—

*Number Boarded* 85.

| — | Total cost | Cost per head |
|---|---|---|
| | £ | £ s. |
| Meat ... ... ... | 620 | 7 6 |
| Bread ... ... ... | 91 | 1 1 |
| Milk... ... ... ... | 146 | 1 14 |
| Fish and poultry ... ... | 193 | 2 5 |
| Vegetables ... ... ... | 124 | 1 9 |
| Butter, eggs, groceries, etc. | 419 | 5 0 |

The averages for the household, consisting of patients, officers and servants, are less interesting, but are quoted for comparative purposes:—

| | |
|---|---|
| Average of occupied beds ... ... ... | 168 |
| Inmates other than patients ... ... ... | 41 |
| Total inmates ... ... ... ... | 209 |

In estimating the cost per bed the provisions of the nursing staff have been included wherever possible.

| — | Cost of hospital household | Cost per head, hospital household | Cost per occupied bed, including nursing staff |
|---|---|---|---|
| | £ | £ s. | £ s. |
| Meat ... ... | 1,134 | 5 8 | 10 8 |
| Fish, poultry, etc. ... | 437 | 2 1 | 3 15 |
| Butter, cheese, etc. | 304 | 1 9 | — |
| Eggs ... ... | 175 | 0 16 | — |
| Milk ... ... | 593 | 2 16 | 4 8 |
| Bread, flour, etc. ... | 188 | 0 18 | 1 13 |
| Grocery ... ... | 326 | 1 11 | — |
| Vegetables ... ... | 206 | 1 0 | 2 0 |
| Malt liquor ... | 67 | 0 6 | — |
| Ice and mineral waters ... | 69 | — | 0 8 patients only |
| Wine and spirits ... | 150 | — | 0 17 patients only |

An analysis of the meat bill showed that in an average week the cost of meat for the officers' table (12 in number) was £5. If this rate were the usual one, which there is reason to suppose, it works out at an average cost per head for this item of £21 a year, the corresponding cost per head for the nursing staff being £7 6s. a year, and for patients and servants together, £4 8s.

On an average day, 84 patients were on meat diet, at an average cost of 3⅞d. each. If this average were maintained through the year, the average cost of meat per occupied bed for *patients only* would work out at £2 17s. a year. But it would not be safe to deduce this from the record of one day. The patients' diet is, in fact, the strand which is most difficult to settle by averages, depending as it does not merely on the class of case under treatment, but on the idiosyncrasies of those responsible for the ordering of extras. Nothing but a complete severance in the accounts of all provisions supplied to the patients can ever be considered a satisfactory method of reckoning the cost of the diets.

The ice and mineral waters are not made in the hospital.

There is a private laundry, but no board is supplied to the laundry-maids.

